# Intracardiac thrombus in Behçet’s disease: a rare case in Morocco

**DOI:** 10.11604/pamj.2020.36.92.23741

**Published:** 2020-06-15

**Authors:** Younes Amchich, Nezha Reguig, Abdelhalim Boucaid, Reda Belghoule, Adil Zegmout, Jamal Eddine Bourkadi

**Affiliations:** 1 Pathology Department, Mohammed VI University Hospital, Oujda, Morocco; 2 Department of Pneumology, Moulay Youssef Hospital, Rabat, Morocco

**Keywords:** Behçet’s disease, pulmonary embolism, immunosuppressive agents, intracardiac thrombus, hemoptysis

## Abstract

Behçet’s syndrome is a systemic inflammatory disease generally presented with the triad of uveitis, oral and genital ulcers. However, it may present with gastrointestinal, central nervous system, skin and vascular disease manifestations. Intracardiac thrombus is a rare but serious complication of Behçet’s disease. A 16-year-old man with Behçet’s syndrome was hospitalized into our department with a history of cough, fever, chest pain, hemoptysis, and weight loss. Transthoracic echocardiography and chest scan revealed a right ventricular thrombus. After one month of treatment with cyclophosphamide, and corticosteroid the intracardiac thrombus has been resolved.

## Introduction

The Behçet’s syndrome is known as a multisystem disorder that affects mainly young adults in Mediterranean, Middle Eastern, and Far Eastern countries. Because there is no laboratory test for Behçet’s syndrome, the diagnosis is very difficult; however, characteristic clinical features such as urogenital apathies, ocular and skin lesions, arthritis, also the neurologic, gastrointestinal, vascular, and pulmonary symptoms aid in the diagnosis. Various cardiovascular manifestations, such as: pancarditis, acute myocardial infarction, conduction system disturbances, and valvular diseases, have been reported but are rare. Intracardiac thrombus formation, as seen in our patient, is exceptional even among cardiovascular cases of Behçet’s syndrome. We report a case of right ventricular thrombus that reveals the Behçet’s syndrome.

## Patient and observation

A previously healthy 17-year-old male with noncontributory past medical history, presented with a one month of dry cough and intermittent hemoptysis. For several weeks prior to hospitalization, he experienced severe exertion dyspnea that limited his mobility. The patient also complained of high fever and shivers in 15 days. The patient acknowledged oral and genital aphtous ulcers occasionally in the past two years. On clinical examination, the patient was alert, oriented, and appeared weak and thin. He weighed 45 kilograms of unintentional weight loss over the preceding two months. The patient vital signs were as follows: temperature 39; blood pressure 128/70 mmHg; heart rate 117 bpm; respiratory rate 20 bpm and an oxygen saturation 97% on room air. He had folliculitis in his face otherwise; the rest of the examination was unremarkable (no oral lesions or penile ulcers objected).

**Figure 1:**
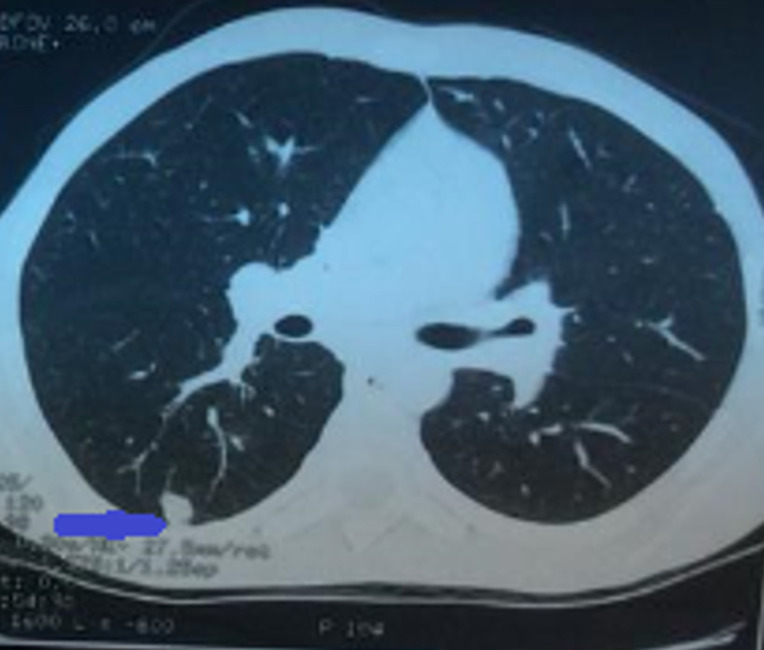
transversal (axial) CT scan of the chest showing condensation in the apical segment of the inferior right lobe

The patient’s white blood count was 10.14 103 cells/ml with normal differential; red cell count was 9.18 GM/dL; hematocrit, 38.5% and platelets were 212 103 cells/ml. C-reactive protein was 105 mg.L-1.The liver function tests, urea and electrolytes were normal. His chest radiography showed right basal consolidation. The three acid-fast bacillus smears were negative, culture was underway. An initial chest CT found condensation in the apical segment of the inferior right lobe (Figure 1). The patient was placed under antibiotic treatment with amoxicillin + clavulanic acid, but the evolution was marked by the non-clinical improvement and persistence of hemoptysis.

**Figure 2:**
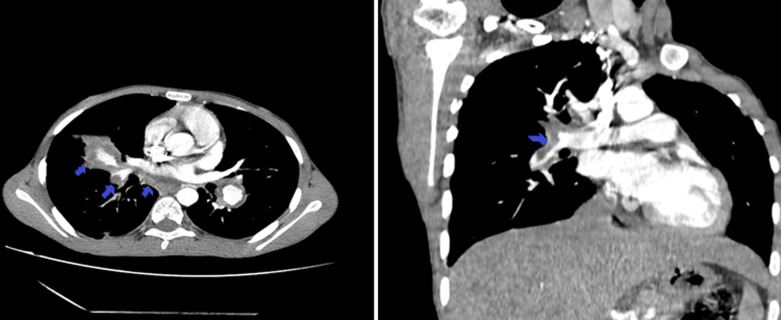
transversal and coronal CT angiography of chest showing pulmonary embolism and right ventricular thrombus

Taking into consideration, the young age of the patient, the history of oral and genital aphthous stomatitis, and the recurrent hemoptysis, a chest CT angiography was requested. It showed a pulmonary embolism of the right pulmonary artery and the right lower lobe bronchus, the presence of a bilateral partial thrombosis ectasia of the right middle lobe bronchus and the left lower lobe brochus and a right intraventricular thrombus (Figure 2). The transthoracic echocardiography showed severe right ventricle enlargement and a 40 mm 26 mm friable mass with a broad attachment to the right ventricular outflow tract and distal right ventricle. The pathergy test was negative. He was finally diagnosed as Behçet's disease (BD) associated with extensive venous thromboembolism including intracardiac thrombus in the right ventricle and pulmonary embolism. The patient was initially treated by anticoagulant then immunosuppressant: methylprednisolone bolus, cyclophosphamide and colchicine. The clinical evolution was favorable.

## Discussion

Intracardiac thrombus is an uncommon complication of Behçet’s disease. The first autopsy revealing intracardiac thrombus was first described in 1977 by Buge *et al*. [1] Since this publication, about fifty similar cases have been registered worldwide which confirm the extreme rarity of intracardiac thrombus [2]. Classic cardiac manifestations, like pericarditis and endocarditis, are exceptional during BD and occur in 1 to 8% of cases [3, 4]. A genetic predisposition of intracardiac thrombus is incriminated since it occurs predominantly in patients from the Mediterranean basin and the Middle East [2, 5]. They have been published approximately 93 cases of Behçet’s disease associated with intracardiac thrombus, most of them are case reports and case series, which have been recently reviewed by Aksu and Tufekcioglu [2] with a male to female ratio of 23:2 and a mean age of 27 years. At the time of detection of ICT, fever, hemoptysis, dyspnea, and cough were the predominant symptoms [2, 6].

In 40-56% of patients with intracardiac thrombus Pulmonary artery involvement was detected, 42-56% of them had venous thrombosis, and 52-55% had [2, 6] pulmonary embolism. Cardiac involvement was the first clinical manifestation of Behçet’s disease in 40-50% of cases [2, 7, 8]. The similarity and difference of the research findings between our case report and that published by other studies are summarized in Table 1 [2]. As previously described [9], intracardiac thrombus mostly involved the right heart, this might partly attributed to extending of thrombi in vena cava and lower pressure of the right heart. Typical echocardiographic features of ICT in BD included the following: involving ventricle rather than atria and mostly in the right heart; often multiple; usually hyper echoic and homogeneous with well-demarcated borders and usually immobile with a broad-based attachment to atria or ventricle. Hence, the diagnosis of Behçet’s disease should be considered when a young male present with intracardiac mass and vascular lesions.

**Table 1:** clinical characteristics and vascular involvement of the previous cases (asterisk) of Behçet’s disease with intracardiac thrombosis in comparison with our case

Items	Previous cases (n = 93)		Our case
Male to female ratio	81:12		Male
Age at diagnosis (years)	27	[mean]	16
Oral involvement (n, %)	91	[98%]	+
Genital involvement (n, %)	86	[93%]	+
Skin involvement (n, %)	45	[57%]	+ [papulopustular lesions]
Pathergy test (n, %)	41	[61%]	negative
Ocular involvement (n, %)	18	[23%]	-
Pulmonary thromboembolism (n, %)	52	[56%]	+
Venous thrombosis (n, %)	39	[42%]	-
- Lower extremity Deep veins	31	[33%]	-
- Inferior vena cava thrombosis	6	[6%]	-
- Superior vena cava thrombosis	10	[11%]	-
Arterial involvement (n, %)	35	[38%]	+ [dilation of the aortic root]
- Pulmonary artery aneurysm	33	[35%]	+
Thrombosis in the right side of the heart	95%		+
- Thrombosis in the right ventricle	74%		+
- Thrombosis in the right atrium	40	[43%]	-
Sinus thrombosis (n, %)	6	[6%]	-
Budd-Chiari (n, %)	7	[7%]	-

* Adapted from Aksu et al. [7]

Immunosuppressant with or without glucocorticoids, are essential in the management of vascular involvement in Behçet’s disease. It has been shown to reduce the relapse rate and to prolong survival in several retrospective studies. Life-threatening conditions such as pulmonary artery involvement, Budd-Chiari syndrome and peripheral arterial aneurysms/occlusions are managed with aggressive medical treatment, including cyclophosphamide and glucocorticoid pulses. Corticosteroids, azathioprine, cyclosporine A, and cyclophosphamide are recommended in the management of acute deep vein thrombosis. In resistant cases, anti-tumor necrosis factor [TNF] agents could be also effective [10]. Whether to add anticoagulants to prevent relapses has been an issue of debate. Several retrospective studies showed the inefficacy of anticoagulation alone or added to immunosuppressant in preventing recurrences [10]. Anticoagulation could increase the risk of aneurysmal rupture [10]. Nevertheless, the tolerance of anticoagulation therapy was satisfactory in patients with low-risk of bleeding after ruling out pulmonary artery aneurysms and it could be used in refractory venous thrombosis along with monoclonal TNF-alpha antagonists. When indicated, surgical treatment is not advised in the active phase of the disease. Invasive arterial techniques may cause pseudo aneurysms, especially in the presence of active inflammation.

## Conclusion

In conclusion, we present a case of intracardiac thrombus reveals Behçet’s disease clinically manifested by prolonged high fever of unknown origin and hemoptysis, successfully treated with immunosuppressant, corticoids and anticoagulant. Different treatment options for intracardiac in Behçet’s disease have been used including steroids, immunosuppressant, anticoagulation and surgery, with different outcomes. Surgical treatment of intracardiac thrombus is usually not recommended. Further studies are needed to guide the management of vascular involvement and other life-threatening complications of Behçet’s disease.
